# Discoidin domain receptor inhibitor DDR1-IN-1 induces autophagy and necroptotic cell death in malignant peripheral nerve sheath tumor

**DOI:** 10.1038/s41420-025-02367-2

**Published:** 2025-03-01

**Authors:** Guan-Yi Lai, Yu-Cheng Lee, Hao-Jui Weng, Kuei-Hung Lai, Min-Chen Hsiang, Kai-Yu Hsu, Chung-Ping Liao

**Affiliations:** 1 Graduate Institute of Medical Sciences, College of Medicine, Taipei Medical University, Taipei, 11031 Taiwan; 2 Graduate Institute of Clinical Medicine, College of Medicine, Taipei Medical University, Taipei, 11031 Taiwan; 3 Department of Dermatology, Taipei Medical University-Shuang Ho Hospital, New Taipei City, 23561 Taiwan; 4 Department of Dermatology, School of Medicine, College of Medicine, Taipei Medical University, Taipei, 11031 Taiwan; 5 International Ph.D. Program in Cell Therapy and Regenerative Medicine, College of Medicine, Taipei Medical University, Taipei, 11031 Taiwan; 6 Graduate Institute of Pharmacognosy, College of Pharmacy, Taipei Medical University, Taipei, 11031 Taiwan; 7 PhD Program in Clinical Drug Development of Herbal Medicine, College of Pharmacy, Taipei Medical University, Taipei, 11031 Taiwan; 8 Traditional Herbal Medicine Research Center, Taipei Medical University Hospital, Taipei, 11031 Taiwan; 9 Cancer Research Center, Taipei Medical University Hospital, Taipei, 11031 Taiwan; 10 Cell Physiology and Molecular Image Research Center, Wan Fang Hospital, Taipei Medical University, Taipei, 11696 Taiwan

**Keywords:** Sarcoma, Autophagy, Necroptosis

## Abstract

Malignant peripheral nerve sheath tumor (MPNST) is a soft tissue sarcoma commonly associated with the tumor-predisposition disorder neurofibromatosis 1. The extracellular matrix collagens contribute to many fibrotic tumors; however, the role of collagen signaling in MPNST was unclear. This study investigated the effects of blocking the interaction between collagens and their receptors in MPNST. We first analyzed the expressions of collagen family proteins in MPNSTs and found an overall increase compared to neurofibroma. Treatment of DDR1-IN-1, a small molecule inhibitor for the collagen receptor discoidin domain receptor, induced a robust MPNST cell death, highlighting the dependence of MPNST survival on collagen signaling. DDR1-IN-1 induced MPNST cell death by activating autophagy and necroptosis signaling. Treatment of necroptosis inhibitors necrostatin-1 or necrosulfonamide reduced the numbers of DDR1-IN-1-induced necrotic cells and autolysosomes, suggesting that the autophagic process depends on necroptosis activation. Combinations of DDR1-IN-1 with other anti-MPNST agents revealed synergistic activities against MPNST. In summary, this study discovered a critical MPNST death signaling induced by the small molecule DDR1-IN-1, which might shed light on future MPNST therapeutic strategies.

## Introduction

Malignant peripheral nerve sheath tumors (MPNSTs) are soft tissue sarcomas with poor prognoses [1]. Around half of the MPNSTs are associated with patients with the genetic disorder neurofibromatosis type 1 (NF1); the non-NF1 cases are commonly associated with radiotherapy. All MPNST subtypes share a similar mutation profile; common mutated genes in MPNSTs are *NF1*, *SUZ12*, *EED*, *CDKN2A*, and *P53* [2–4].

MPNSTs in NF1 patients are transformed from benign neurofibromas. The tumor tissue of neurofibromas is characterized by dense collagens, which take about 30-50% lipid-free dry weight of the tumor [5]. Histological characterization of these collagens revealed type I, III, IV, and V collagens [5]. Other extracellular matrix (ECM) molecules, such as fibronectin and laminin, are also noticed in the neurofibroma microenvironment [5, 6]. A recent single-cell sequencing further revealed that the pro-tumorigenic collagen VI is also a dominant ECM in human cutaneous neurofibroma [7]. Although abundant ECM deposition is a well-recognized feature in neurofibroma, the role of ECM in its associated malignancy MPNST is unclear.

MPNST ECM structure could be related to genetic mutation status. *SUZ12* and *EED* are commonly mutated genes in MPNSTs. SUZ12 and EED are key components of polycomb repressive complex 2 (PRC2), a histone methyltransferase for histone H3 lysine 27. PRC2 loss in MPNSTs (e.g. SUZ12/EED mutations) induces an epigenetic switch from H3K27me3 to H3K27Ac, allowing a relaxed chromatin status for gene expressions. Intriguingly, a mouse MPNST model with PRC2 loss revealed an increase in MPNST fibrosis and lung metastasis through modulating the expression of ECM remodeling enzyme families MMP and LOX [8]. In addition, human MPNST samples with PRC2 mutations showed increases in collagen genes COL1A1 and COL1A2 expression [8].

In human MPNST, a clinical pathology analysis of 17 MPNST cases showed strong expression of integrin β3, moderate expression of integrin α6 and laminin, and weak expression of collagen IV, fibronectin, and vitronectin [9]. Notably, collagen IV and laminin expressions were lower in the tumor than in the adjacent neoplastic nerve tissue.

In recent years of cancer research, the importance of ECM has been recognized beyond the structure of tumors. ECM could be critical for tumor cell progression and metastasis [10]. Neurofibromas are collagen-rich tumors, and their malignant form MPNSTs are associated with ECM remodeling during transformation [5, 8]. However, whether ECM plays a role in sustaining these *NF1* mutant tumor proliferation and survival is not clear.

We are interested in the contribution of collagen signaling to MPNST cancer cell proliferation and survival. Extracellular collagens can bind to membrane receptor integrin, discoidin domain receptor (DDR), glycoprotein VI (GPVI), and leukocyte-associated immunoglobulin-like receptor-1 (LAIR-1) [11]. In the context of cancers, microenvironmental collagens are likely to activate cancer cell growth through the receptor of DDR1, as it is associated with the cell proliferation signaling molecules RAS, PI3K, NFκB, and STAT [12].

DDR1 has a binding affinity to collagens I II, III, and IV [13]. DDR1 expressed on tumor cells modulates collagen fiber arrangement and manipulates microenvironmental immunity [14]. DDR1 controls tumor cell proliferation and metabolism, and DDR1 expression level correlates with patient survival and response to therapy [15]. Therefore, inhibiting DDR1 signaling by small molecules has been proposed as an intervention for cancers [16].

DDR1-IN-1 is a selective DDR1 inhibitor with an IC50 of 105 nM [17]. DDR1-IN-1 inhibited melanoma proliferation in vitro and in vivo [18]. It also suppressed oral squamous cell carcinoma cell growth and stemness [19]. In addition, DDR1-IN-1 can also inhibit hepatocellular carcinoma metastasis by downregulating genes for epithelial-mesenchymal transition [20]. In an in vitro experiment, DDR1-IN-1 inhibits DDR1 autophosphorylation in the presence or absence of collagen stimulation [17], suggesting a baseline DDR1 activation without exogenous collagen stimulation.

In this study, we employed DDR1-IN-1 to explore the contributions of DDR1 signaling to MPNST proliferation and survival. Our data showed that DDR1-IN-1 induced MPNST cell death by activating autophagy and necroptosis. This finding suggested that ECM-mediated signaling could be a potential therapeutic approach against MPNST. Importantly, our data revealed that DDR1-IN-1 is a novel necroptosis inducer. In addition, combinations of DDR1-IN-1 with chemotherapeutic agents, MEK inhibitors, or SHP2 inhibitors induced synthetic lethality in MPNST cancer cells.

## Results

### Collagen receptor DDR inhibitor DDR1-IN-1 induced abrupt cell death in MPNST

**Fig. 1:**
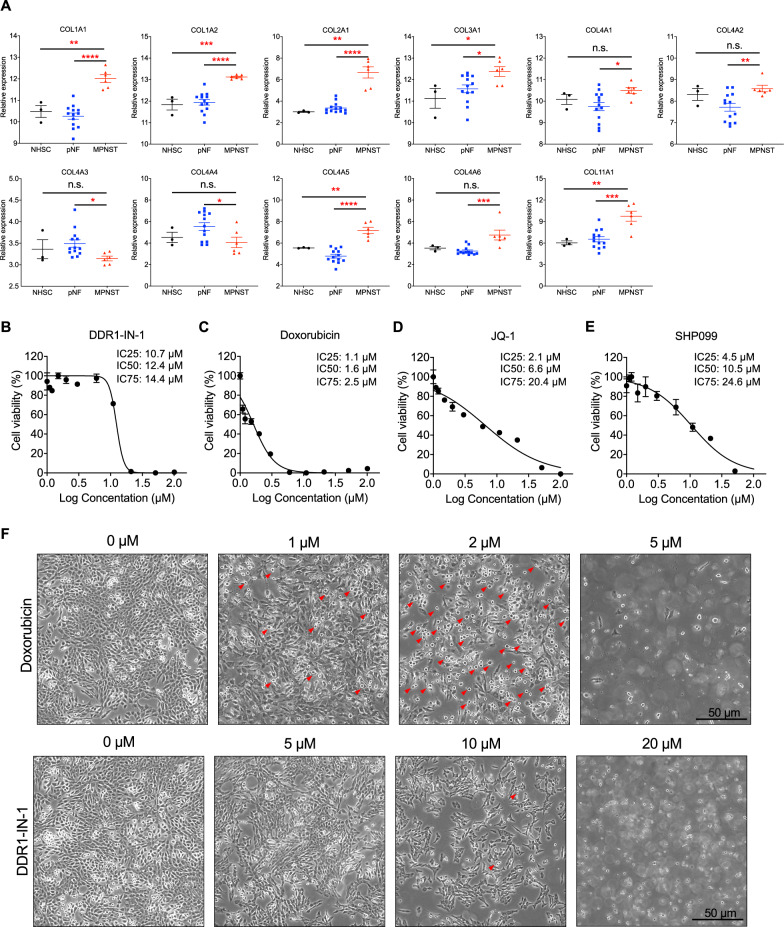
DDR1-IN-1 induced MPNST cell death. **A** Collagen gene expressions of nerve (NHSC), neurofibromas (pNF), and MPNST from GSE41747-10371 were analyzed and plotted. The *p* value was calculated and demonstrated. *P < 0.05, **P < 0.01, ***P < 0.001, ****P < 0.0001. **B–E** To identify potential inhibitors that perform anti-MPNST activity, we introduced 3 inhibitors and 1 clinical-used anti-cancer drug to an MPNST cell line, S462. 0-100 μM DDR1-IN-1, Doxorubicin, JQ-1 and SHP099 were introduced to cells for 48 hours. Cell viability was evaluated through the MTT assay, and the IC25, IC50, and IC75 were calculated (*n* = 4). **F** Images of cells treated with indicated doses of Doxorubicin and DDR1-IN-1 for 24 hours were shown.

To explore potential collagen signaling in MPNSTs, we utilized the previously published transcriptome dataset (GSE41747-10371) [21] to delineate the mRNA expressions of collagen family members in control nerve, plexiform neurofibroma, and MPNST. By comparing the groups of MPNST with neurofibroma, the results revealed significant increases in 9 out of 11 genes (COL1A1, COL1A2, COL2A1, COL3A1, COL4A1, COL4A2, COL4A5, COL4A6, COL11A1), and decreases in 2 out of 11 genes (COL4A3, COL4A4) (Fig. 1A), highlight a role of collagens during MPNST transformation. We also analyzed the expressions of collagen receptors and ECM remodeling enzyme MMPs (Suppl. Figure 1), but the data did not show a clear trend as collagens.

In addition to clinical data, we also analyzed the main collagen receptor DDR1 protein expression in four human MPNST cell lines. Our results showed that DDR1 protein levels were significantly higher in MPNST cells than in control Schwann cells (Supplemental Fig. 2A). At present, we do not have a certain answer for the differential DDR1 mRNA and protein expressions in different MPNST samples (Supplemental Fig. 1 and Supplemental Fig. 2A), a possible explanation might be post-transcriptional regulation. However, the activation of DDR1 signaling is expected to occur in both tumor and cell line samples as either the increases of collagen ligands or the DDR1 receptor should activate DDR1 signaling. In addition, to ascertain that extracellular collagen can trigger DDR1 signaling in MPNST, two MPNST cell lines were treated with collagen, followed by the analysis of DDR1 activation. The phosphorylated DDR1 at Y792 was significantly increased upon collagen stimulation (Supplemental Fig. 2B). Our data collectively demonstrated the activation of DDR1 signaling in MPNSTs.

To determine whether MPNST collagens and DDR1 signaling play a role in MPNST proliferation survival, MPNST S462 cells were treated with pan-DDR inhibitor DDR1-IN-1 followed by MTT assays. The MTT assay evaluates the overall viable cell number by determining their metabolic activity. DDR1-IN-1 showed potent activity to inhibit cell viability with an IC50 of 12.4 µM (Fig. 1B). Intriguingly, when we analyzed the MTT data, we noticed an unusual curve in which DDR1-IN-1 affected S462 cell viability drastically within a very narrow range of drug concentration changes. This is very different from other anti-MPNST agents, including chemotherapeutic agent doxorubicin (Fig. 1C), BET bromodomain inhibitor JQ-1 (Fig. 1D), and SHP2 Inhibitor SHP099 (Fig. 1E); these agents induce apoptotic cell death or arrest cell cycle in MPNST [22–24]. To contrast the differences of DDR1-IN-1 with these agents, we used the ratio of IC75 over IC25 to reflect the trend of cell viability change, the IC75/IC25 for DDR1-IN-1, doxorubicin, JQ-1, and SHP099 are 1.35, 2.27, 9.71, 5.46 respectively (Fig. 1B–E), emphasizing a unique ability of DDR1-IN-1 to induce a sudden overwhelmed cellular stress when over a certain drug concentration.

To further characterize the above activity, we analyzed DDR1-IN-1 treated MPNST at the lethal dose (the minimum concentration causes all cells to die) and the sublethal dose (a concentration degree less than the lethal dose). We also included doxorubicin as a control since it is a well-characterized apoptosis inducer [25]. For doxorubicin, all cells were dead at the lethal dose 5 µM; at the sublethal dose 2 µM, a significant portion of membrane blebbing apoptotic cells was observed (Fig. 1F). In contrast, DDR1-IN-1 induced all cell death at 20 µM; at the sublethal dose of 10 µM, only a very small number of cells showed blebbing membrane, lacking classical apoptotic signatures. Our results suggested that DDR1-IN-1 induced a special form of cell death other than apoptosis, and we were very interested in exploring this DDR1-IN-1-induced cell death in MPNST.

### DDR1-IN-1 induced LC3B-II accumulation in MPNST

**Fig. 2:**
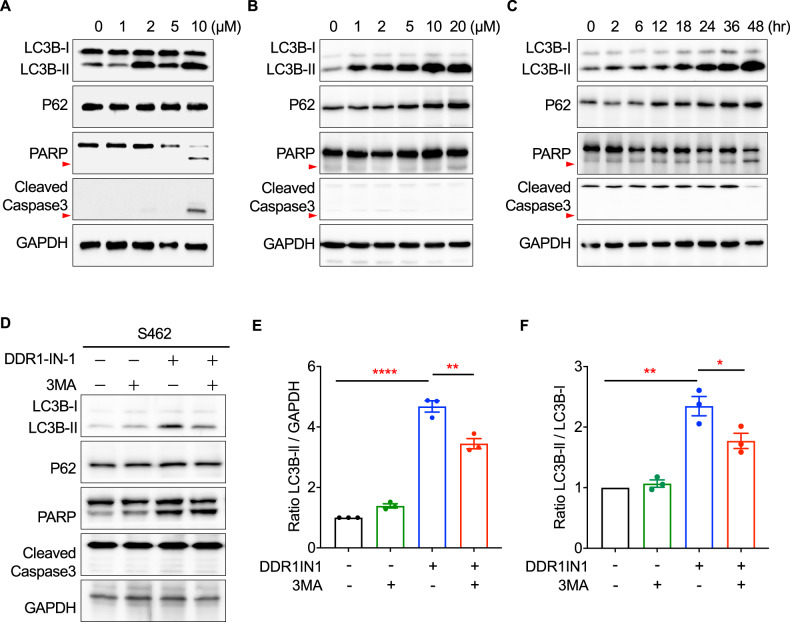
DDR1-IN-1 resulted in the accumulation of LC3B-II in MPNST cells. The total protein lysates of (**A**) S462 and (**B**) STS26T MPNST cells treated with 0-10 μM DDR1-IN-1 for 24 hours, as well as (**C**) STS26T cells treated with 10 μM DDR1-IN-1 for 0-48 hours were extracted. Western blotting was performed to reveal the expression levels of various cell death-related proteins that participated in the autophagy or apoptosis pathway. The red arrowheads point out the cleaved PARP. **D** Autophagy inhibitor, 3-MA (5 mM), was co-treated separately with 10 μM DDR1-IN-1 to S462 cells for 24 hours. Total protein was extracted and the cell death-related markers were demonstrated through western blotting. The cleaved-Caspase3 is pointed out by the red arrow. **E** The ratio of LC3B-II to GAPDH was calculated and compared between each group (*n* = 3), and (**F**) the ratio of LC3B-I to LC3B-II was calculated and compared between each group (*n* = 3). The ratio is plotted in bar chart and the *p* values are shown. *P < 0.05, **P < 0.01, ***P < 0.001, ****P < 0.0001.

DDR1-IN-1 was previously shown to induce autophagic cell death and to increase radiochemosensitivity in glioblastoma [26]. We, therefore, investigated whether DDR1-IN-1 also activated autophagic signaling in MPNST. During autophagy, cytosolic LC3-I conjugates to phosphatidylethanolamine to form LC3-II, and the level of LC3-II is a common marker to evaluate autophagy [27]. Our results showed that DDR1-IN-1 induced significant LC3B-II accumulation in two MPNST cell lines (S462 and STS26T) beginning from a low concentration (2 and 1 µM) in a dose-dependent manner (Fig. 2A, B), confirming the activation of autophagy. Intriguingly, P62 (SQSTM1) was very little affected or slightly increased (Fig. 2A, B), but the mechanism is unclear. Importantly, we also analyzed apoptosis markers PARP and caspase-3 cleavage, and our data showed that these apoptosis signatures were either not detected or only detected at the highest concentration condition, virtually excluding the role of apoptosis in DDR1-IN-1-induced MPNST cell death (Fig. 2A, B). In addition, the time-dependent experiment also revealed a similar trend of the above signature changes (Fig. 2C).

To ascertain the LC3B-II changes mentioned in the previous paragraph were caused by an induction of autophagy, PI3K inhibitor 3-Methyladenine (3-MA) was used to treat MPNST together with DDR1-IN-1. We used the ratios of LC3B-II/GAPDH and LC3B-II/LC3B-I based on the Western blotting densitometry analysis to determine the DDR1-IN-1 and 3-MA activity. These ratios are commonly used methods to determine autophagy activation [27]. Our data revealed that 3-MA reduced the levels of DDR1-IN-1-induced LC3B-II (Fig. 2D–F), demonstrating the ability of DDR1-IN-1 to induce autophagy. Another similar experiment was performed by VPS34 inhibitor SAR405, which also showed a similar result (Suppl Fig. 3). Taken together, our data validated DDR1-IN-1 as an autophagy inducer in MPNST, and this autophagic cell death did not or minimally involve apoptosis.

Since DDR1-IN-1 induced cell viability change in Fig. 1B can also be possibly controlled by inhibition of cell proliferation signals, we further analyzed markers for cell proliferation signaling, including mTOR-, ERK-, and CDK-related proteins, and we did not detect a significant change in these markers in response to DDR1-IN-1 treatment (Suppl Fig. 4).

### DDR1-IN-1 increased the abundance of autolysosomes in MPNST

**Fig. 3:**
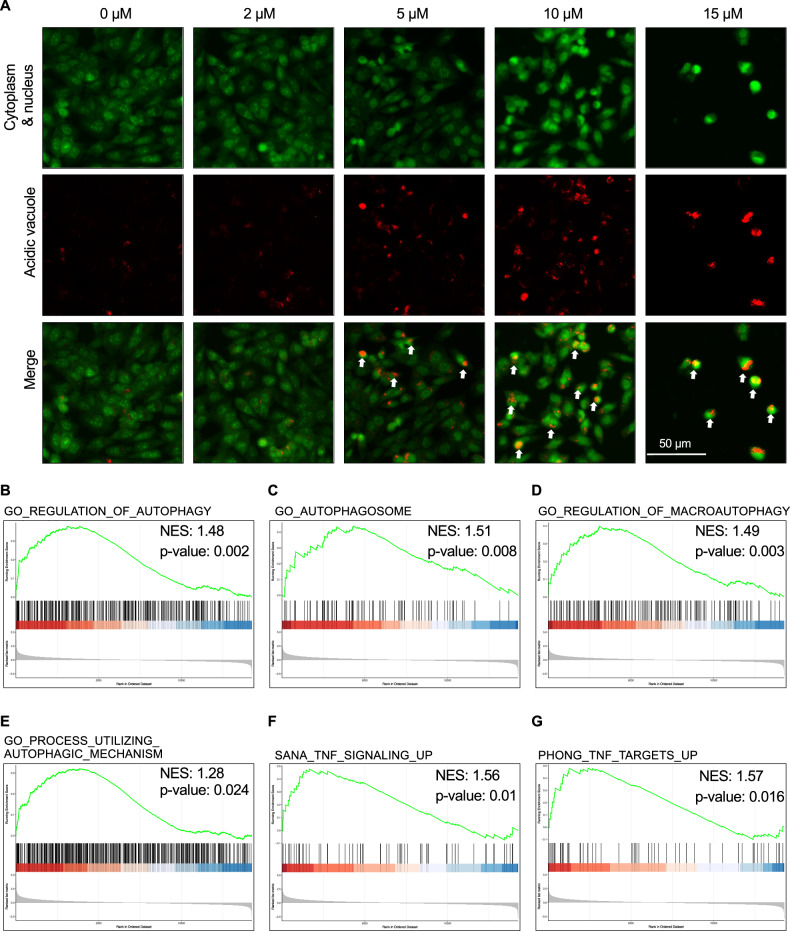
DDR1-IN-1 induced autophagy in MPNST. The autophagy activity was visualized through the Acridine Orange (AO) assay (**A**). The fluorescent images represent the S462 cells under AO assay in response to 0–15 μM DDR1-IN-1 treatment for 24 hours. Green fluorescent represents the cytoplasm and nucleus; red fluorescent indicates the acidic vacuoles and the white arrows point to the cells undergoing autophagy. Transcriptomic data are also presented. **B**–**G** RNAs were collected after treating S462 cells with 10 μM DDR1-IN-1 for 24 hours. The RNA-seq was performed and the data was analyzed (*n* = 3). GSEA analysis data represents the correlation between DDR1-IN-1-treated MPNST cells and autophagy.

During autophagy, LC3-II is recruited to autophagosomal membranes. The autophagosomes then fuse with lysosomes to form autolysosomes, and the internal components are degraded by lysosomal enzymes [28]. Autolysosomes are acidic vesicular organelles that can be identified by the cell-permeable fluorophore acridine orange [29]. Acridine orange stains the whole cell green and the acidic vesicular organelles (e.g., autolysosome) orange to red. Our results revealed that the DDR1-IN-1 induced the accumulation of autolysosome (Fig. 3A), confirming the activation of autophagy, and the data is also in line with our finding of LC3B-II accumulation in Fig. 2.

To comprehensively characterize the downstream signaling pathways and molecules responding to DDR1-IN-1 treatment, we performed RNA-seq analysis in S462 cells with 10 µM DDR1-IN-1 treatment for 24 hr. As expected, multiple gene sets related to autophagy or autophagosome were correlated to DDR1-IN-1 treatment (Fig. 3B–E). Collectively, our experimental data highlighted the activation of autophagic signaling under DDR1-IN-1 treatment in MPNST.

Interestingly, we noticed that the GSEA analysis also revealed positive correlations of DDR1-IN-1 treatment with tumor necrosis factor (TNF) signaling (Fig. 3F) and TNF targets (Fig. 3G). TNF is a central regulator of apoptosis and necroptosis [30]. By the data in Fig. 2A–C, we excluded the role of apoptosis in DDR1-IN-1-induced MPNST cell death; therefore, we were interested to know whether DDR1-IN-1 can induce necroptosis in MPNST.

### DDR1-IN-1 induced necroptotic cell death in MPNST

Necroptosis is a type of programmed cell death that undergoes a necrotic process, and it is distinct from the cell death caused by apoptosis or autophagy. To investigate whether necroptosis is activated during DDR1-IN-1 treatment, we performed Annexin V (AV) and propidium iodide (PI) double staining in DDR1-IN-1 treated MPNST cells. AV has a high affinity for phosphatidylserine, which translocates from the inside to the outside of the plasma membrane during apoptosis. PI binds to DNA by intercalating between the nucleotide bases; since PI is membrane impermeable, it stains necrotic or late apoptotic cells with compromised membrane integrity.

**Fig. 4:**
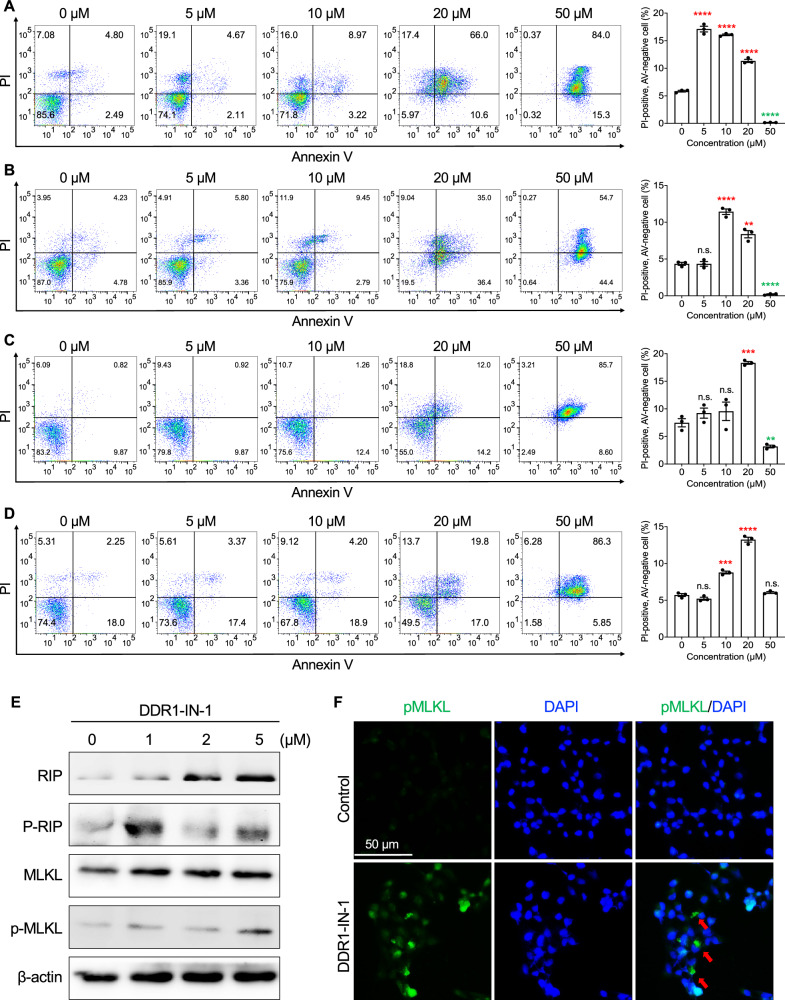
DDR1-IN-1 induced necroptotic cell death in MPNST. AV/PI double staining was performed in different MPNST cell lines, including S462 (**A**), ST8814 (**B**), STS26T (**C**), and T265 (**D**). The MPNST cells were treated with 0-50 μM DDR1-IN-1 as indicated for 24 hours, the AV/PI double staining was manipulated as Materials and Methods described, and the fluorescent intensity was detected through flow cytometry (*n* = 3). The PI-positive cell (%) was plotted in the bar chart and the p-value was shown. *P < 0.05, **P < 0.01, ***P < 0.001, ****P < 0.0001. **E** Total protein was extracted after treating ST8814 cells with 0-5 μM DDR1-IN-1 for 24 hours and the necroptosis-related markers were evaluated. **F** The immunofluorescent images of control and DDR1-IN-1 treated S462 cells are shown. The green fluorescent represents the p-MLKL, and the red arrows indicate the cells undergoing necroptotic cell death.

In the AV + PI staining, we focused on the PI + /AV- population and regarded them as early necrotic cells. In the DDR1-IN-1-induced cell death, we observed a significant increase in the PI + /AV- population in four MPNST cell lines (Fig. 4A–D), pointing to a necrotic cell death. In addition, the AV + /PI- population seemed to be minimally affected, suggesting a non-apoptotic cell death. Of note, among the four tested MPNST cell lines, S462 demonstrated the most classical necrotic signatures (Fig. 4A).

To further determine whether DDR1-IN-1 induced necrotic cell death through programmed signaling, markers for necroptosis were evaluated, including RIP, p-RIP, MLKL, and p-MLKL. Our results revealed that the protein levels of RIP, MLKL, and p-MLKL were increased during DDR1-IN-1 treatment in S462 MPNST cells, confirming the activation of necroptosis signaling (Fig. 4E). Furthermore, the immunofluorescence staining also revealed an elevation of p-MLKL levels in DDR1-IN-1-treated S462 MPNST cells (Fig. 4F). Taken together, our data revealed that DDR1-IN-1 is a necroptosis inducer in MPNST cells, which is a novel activity not reported previously.

### Necroptosis inhibitors reversed DDR1-IN-1-induced MPNST cell death

**Fig. 5:**
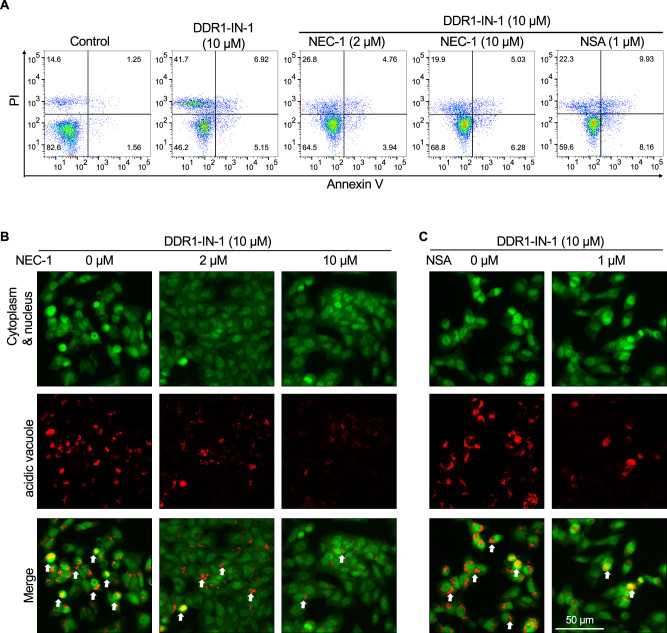
Necroptosis inhibitors reversed DDR1-IN-1-induced MPNST cell death. Necrostatin-1 (NEC-1) and Necrosulfoamide (NSA) are commonly used necroptosis inhibitors in research. MPNST S462 cells are treated with or without 10 μM DDR1-IN-1, combined with NEC-1 or NSA (**A**) in various concentrations as indicated. Annexin V/ PI double staining was performed and the fluorescent intensity was analyzed through flow cytometry. **B, C** AO staining reveals the autophagy level after 10 μM DDR1-IN-1-treated cells exposed to 0–10 μM NEC-1 or 0-1 μM NSA. Green fluorescent represents the cytoplasm and nucleus; red fluorescent indicates the acidic vacuoles and the white arrows point to the cells undergoing autophagy.

To ascertain DDR1-IN-1 activated necroptosis in MPNST, we utilized necroptosis inhibitors necrostatin-1 (Nec-1) and necrosulfonamide (NSA) to confirm our experimental findings. Nec-1 and NSA inhibit the activity of RIP and MLKL, respectively. Our results showed that Nec-1 and NSA reduced the PI + /AV- cell population in DDR1-IN-1 treated MPNST (Fig. 5A), confirming the necroptosis event induced by DDR1-IN-1. Furthermore, we thought it is important to answer whether DDR1-IN-1-induced necroptosis (Fig. 4) and autophagy (Fig. 3) are correlated events. To address this question, we used Nec-1 to treat DDR1-IN-1-induced autophagic cells, and we found that Nec-1 is capable of reducing the abundance of DDR1-IN-1-induced autolysosomes (Fig. 5B). A similar activity was also observed in another necroptosis inhibitor NSA (Fig. 5C). These results together revealed that DDR1-IN-1-induced autophagy is dependent on the necroptosis signaling.

### DDR1-IN-1 induced synergistic cell death with other anti-MPNST agents

**Fig. 6:**
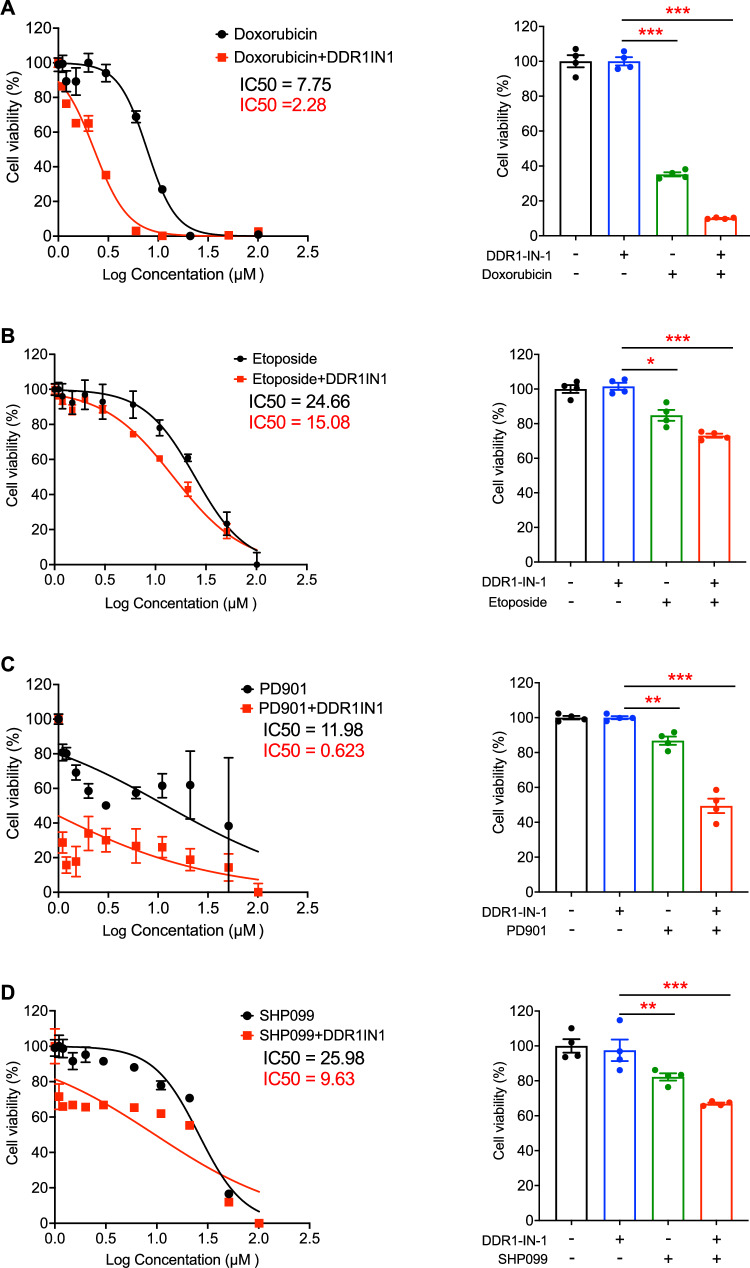
DDR1-IN-1 induced synergistic cell death with other anti-MPNST agents. S462 MPNST cells were treated with 5 μM DDR1-IN-1 combined with 0-100 μM doxorubicin (**A**), etoposide (**B)**, PD0325901 (**C)**, or SHP099 (**D**) for 48 hours. Cell viability was evaluated through the MTT assay and the IC50 was calculated. The cell viability of cells exposed to 5 μM DDR1-IN-1 and/ or 10 μM anti-MPNST agents were plotted as bar charts as indicated, and the p-value was demonstrated (*n* = 3). *P < 0.05, **P < 0.01, ***P < 0.001, ****P < 0.0001.

A common current cancer therapeutic strategy is combining two drugs’ actions on independent targets or pathways, which aims to achieve the goal of synergistic anti-cancer activity [31]. If DDR1-IN-1 is a new necroptosis inducer, as we report here, DDR1-IN-1 may exert an additive or synergistic activity when combined with other anti-cancer agents. To address this theory, we treated S462 MPNST cells with 5 µM DDR1-IN-1 in combination with increasing concentrations of chemotherapeutic agent doxorubicin, etoposide, MEK inhibitor PD0325901, and SHP2 inhibitor SHP099. In our previous experiment, S462 cells treated with 5 µM DDR1-IN-1 showed no sign of death, but cell death was noticed when beyond 10 µM (Fig. 1B, D). Therefore, we set 5 µM DDR1-IN-1 in combination with other anti-MPNST agents. Our data revealed that DDR1-IN-1 further reduced the S462 viability when combined with doxorubicin (Fig. 6A), etoposide (Fig. 6B), PD0325901 (Fig. 6C), and SHP099 (Fig. 6D). Noteworthy, among these combinations, DDR1-IN-1 induced the best synergistic activity with doxorubicin (Fig. 6A). This is important evidence emphasizing that the combination of necroptosis and apoptosis inducers could achieve a highly potent anti-cancer activity.

### DDR1-IN-1 can also induce necroptosis in non-MPNST cancer cells

The induction of necroptosis is a new finding of the cell death mechanism induced by DDR1-IN-1. To determine whether the necroptotic effect is limited to MPNST cells, we treated breast cancer cell line MCF-7 with DDR1-IN-1. Similar to S462 cells, we observed a sudden cell death phenotype (a necroptosis signature) in MCF-7 cells when treated with DDR1-IN-1 (Supplemental Fig. 5A), while doxorubicin induced a classical apoptosis signature. The AV/PI staining further revealed an increased PI + /AV− cell population in MCF-7 cells as a response to DDR1-IN-1 treatment (Supplemental Fig. 5B), a similar phenomenon was also noticed in glioblastoma U-87 cell line (Supplemental Fig. 5C). Importantly, the addition of necroptosis inhibitor NEC-1 attenuated the DDR1-IN-1-induced PI + /AV− cell population (Supplemental Fig. 5C&C), confirming the event of necroptosis induced by DDR1-IN-1 in non-MPNST cancer cells.

## Discussion

**Fig. 7:**
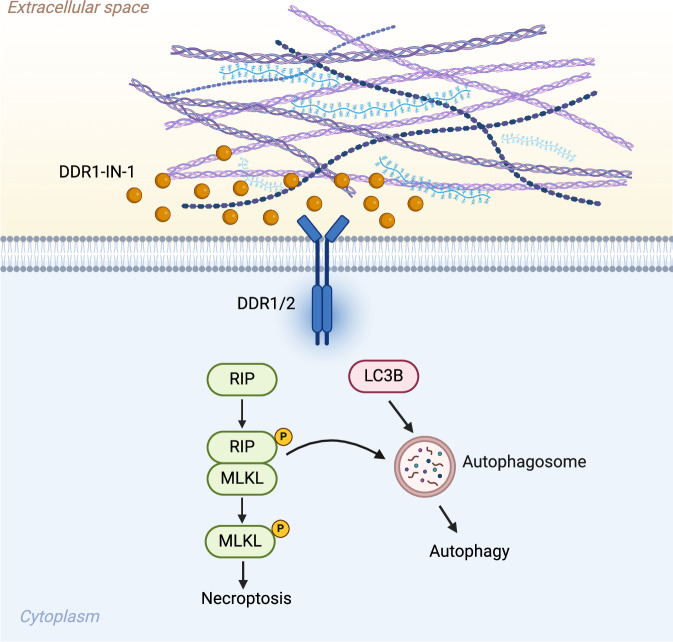
Graphical summary of DDR1-IN-1-induced MPNST cell death. In summary, introducing DDR1-IN-1 to MPNST cells inhibits the collagen-related DDR1/2 signals and performs an anti-MPNST activity. DDRI-IN-1 induces MPNST cell death through autophagy and necroptosis pathway. Under DDR1-IN-1 treatment, the necroptosis signaling also triggers the cell autophagy and enhances the anti-MPNST activity. Created with BioRender.com.

ECM plays a crucial role in cancer progression and metastasis, and it is dynamically regulated by deposition, modification, degradation, and remodeling [10]. Tumor microenvironmental collagens modulate cancer proliferation, metabolism, stemness, angiogenesis, metastasis, drug resistance, and immunity [32]. The change in collagen composition also greatly impacts cancer progression and prognosis [33]. Collagens primarily signal to cancer cells in cancers by binding to the DDR1/2 receptors. MPNST is a soft tissue sarcoma commonly transformed from collagen-rich neurofibromas [5]. In this study, we found that inhibition of DDR1 by DDR1-IN-1 induces necroptosis and autophagy in MPNST (Fig. 7). Additionally, combination of DDR1-IN-1 with chemotherapeutic targeted therapeutic agents induced synergistic MPNST cell death.

The expression of DDR1 is associated with Schwann cell pathology. DDR1 and DDR2 are two of the most significantly repressed genes in plexiform neurofibroma mice after treatment with the kinase inhibitor cabozantinib [34]. DDR1 expression is upregulated in Lats1/2 deficient MPNST mouse model [35]. DDR1 is also a recurrent mutation gene in schwannoma [36]. Interestingly, in our investigation comparing the DDR1 mRNA expression in Schwann cells, neurofibroma, and MPNST, we did not notice any significant difference (Supplemental Fig. 1A); however, the human MPNST cell line analysis revealed increased DDR1 protein expressions (Supplemental Fig. 2A). This discrepancy might suggest a post-transcriptional regulation of DDR1 expression in MPNST.

A recent study revealed that human cutaneous neurofibromas contain abundant collagen VI, but low in collagen I [7]. Another study further found that SOX9 activates collagen VI secretion in neurofibroma through the upregulation of procollagen C-endopeptidase enhancer (PCOLCE) [37]. High SOX9 expression correlates with the malignant potential of neurofibroma/MPNST [38]. Moreover, PRC2 loss drives MPNST matrix remodeling and metastasis [8]. These data suggest that tumor mutational status can change collagen homeostasis in *NF1* mutant tumors.

Inhibition of DDR1 has been shown as an anti-cancer strategy in multiple cancer types [39]. Small molecules of DDR1 generally target the autophagy pathway. However, they can be either pro- or anti-autophagy, depending on the context. DDR1-IN-1 induces autophagic cell death in glioblastoma cells [26]. DDR1-IN-2 promotes chondrocyte autophagy [40]. Nilotinib increases autophagy gene expression [41]. However, blocking DDR1 signaling cascades by an SH2 superbinder inhibited collagen-induced autophagy in pancreatic cancer [42]. DDR1 knockout in vivo decreased osteoblast/osteocyte autophagy [43]. In this study, our results showed that DDR1-IN-1 induced autophagy in MPNST, as evidenced by the accumulation of LC3B-II and autolysosomes.

Compared to apoptosis and autophagy, necroptosis is a relatively uncommon form of cell death. Necroptosis is mostly induced by death receptors, such as FAS and TNF-α receptor [44]. The central molecules mediating necroptosis are RIPK1, RIPK3, and MLKL. In cancers, necroptosis can be pro- or anti-cancer, and the above necroptotic factors can be up- or down-regulated [44, 45], highlighting the sophisticated regulations of necroptosis in cancer development. In this study, we report that DDR1-IN-1 is a novel necroptosis inducer. Only a few agents are known to function as a necroptosis inducer [45]. To our knowledge, DDR1-IN-1 is the first ECM receptor inhibitor ever reported to induce necroptosis. In addition, it was previously noticed that DDR1-/- tumors are more necrotic than DDR1 + /+ and DDR1 + /- tumors [46], which is in line with our findings. Collectively, current evidence suggests a novel connection of collagen receptor DDR to necroptosis, which might lead to a new paradigm of cancer therapy.

## Conclusion

In this study, we demonstrated that targeting the collagen-stimulated DDR1 signaling in MPNST cells by the DDR1 inhibitor DDR1-IN-1 induced autophagic and necroptotic cell death, emphasizing the critical role of ECM signaling in MPNST survival. The activity of necroptosis induction is a new finding of DDR1-IN-1. As a novel necroptosis inducer, DDR1-IN-1 is capable to induce necroptosis in MPNST cell lines S462, ST8814, T265, STS26T, breast cancer cell line MCF-7, and glioblastoma cell line U-87. Combination of DDR1-IN-1 with other anti-MPNST agents induced synergistic cell death effects. Collectively, this study provides important insight on targeting cancer cell survival by activating an uncommon form of cell death, and shed light on future combination therapies again cancers.

## Materials and Methods

### Cell culture and reagents

The MPNST cell line S462 was kindly shared by Dr. Karen Cichowski (Harvard Medical School), and ST8814, STS26T, and T265 were kindly shared by Dr. Nancy Ratner (Cincinnati Children’s Hospital Medical Center). The normal Schwann cell line (CRL-3391), breast cancer MCF7 cell line (HTB-22), and glioblastoma U-87 cell line (HTB-14) were obtained from ATCC (Virginia, USA). All cell lines were cultured in Dulbecco’s Modified Eagle Medium (DMEM) (Gibco, 11995065, New York, USA) supplemented with 10% fetal bovine serum (Cytiva, SH30396.03, Washington, D.C., USA), L-glutamine (Gibco, 25030081), and, 1% Penicillin-Streptomycin (Gibco, 15140122). All the cell lines were maintained in 10-cm cell culture dish in an incubator at 37°C with 5% CO2. The screening for Mycoplasma contamination was performed using the Mycoplasma PCR Detection Kit (Applied Biological Materials, G238, Richmond, Canada). DDR1-IN-1 (MedChemExpress, HY-13979, New Jersey, USA) was resuspended in DMSO and was used to treat MPNST cells as the conditions indicated in the figures. 0 µM represented the cells treated with vehicle only. For collagen stimulation assay, collagen type I (Merck, 08-115, Darmstadt, Germany) was added into culture media at the concentration of 25 µg/mL and incubated for 24 h.

### MPNST transcriptome analysis

The differential gene expressions of collagens, collagen receptors, and ECM remodeling MMPs were analyzed from the online public clinical MPNST database GSE41747-10371. The data include samples of human nerve (*n* = 3), human plexiform neurofibroma (*n* = 13), and human MPNST (*n* = 6). The data were compared using an unpaired two-sided t-test using GraphPad Prism 10 for Mac. The error bars represented SD, and the *p* values < 0.05 were considered as significant differences.

### RNA-sequencing analysis

S462 MPNST cells were treated with 10 µM DDR1-IN-1 for 24 hr, followed by RNA extraction by TRIzol (Invitrogen, 15596018, Massachusetts, USA). Briefly, cells from one well of a 6-well plate were harvested in 1 ml TRIzol reagent, RNA was extracted by the addition of 0.2 ml chloroform, and RNA in the aqueous phase was precipitated by isopropanol. The quality of RNA was determined by the ratio of OD260/280 measured by Nanodrop, while the ratio between 1.8-2.0 was considered to be ideal quality. Qualified RNA samples were subjected to RNA-seq analysis (*n* = 3) provided by BIOTOOLS. The RNA-seq data of DDR1-IN-1 treatment in S462 cells has been deposited in the National Center of Biotechnology Information (NCBI) Sequence Read Archive (SRA), accession number PRJNA1196119.

### Western blotting

MPNST cells were harvested by 0.25% Trypsin-EDTA (Gibco, 25200056), washed by PBS, and pelleted in a 1.5 ml microcentrifuge tube. The cell pellet was resuspended in radioimmunoprecipitation lysis buffer (RIPA buffer), supplemented with protease inhibitors cocktail (MedChemExpress, HYK0010) and phosphatase inhibitor cocktails I&II (MedChemExpress, HYK0021, HYK0022) on ice for 30 min, followed by centrifugation at 1,4000 rpm for 20 mins at 4°C. The protein concentration was determined by the BCA Protein Assay Kits (Thermo Fisher, Pierce 23225, Massachusetts, USA). A total of 20 µg protein was used for each sample of protein electrophoresis. Protein samples were mixed with 5X SDS Loading buffer (Bioman, P1002, New Taipei City, Taiwan) and incubated at 95°C for 10 min. Protein electrophoresis was performed by the Mini-PROTEAN Tetra Vertical Electrophoresis Cell at 140 volts for 1.5-2 h and the proteins were transferred onto the polyvinylidene fluoride membrane (Millipore, IEVH85R, Massachusetts, USA) by 75 volts for 1.5 hours. Blots were blocked by 2% bovine serum albumin in PBS at RT for 1 hour.

To detect a specific protein, a blot was incubated with a primary antibody by shaking at 4°C overnight. The antibodies used in this study were: β-actin (Cruz, sc-47778, Texas, USA), GAPDH (Santa Cruz, sc-32233), Phospho-ERK (Tyr 204) (Santa Cruz, sc-7383), Erk1/2 (Cell Signaling, 4695, Massachusetts, USA), PARP (Cell Signaling, 9542), LC3B (Cell Signaling, 2775), Phospho-RIP (Ser166) (Cell Signaling, 65746), RIP(Cell Signaling, 3493), Phospho-MLKL (Ser358) (Cell Signaling, 91689), MLKL (Cell Signaling, 14993), Cleaved Caspase-3 (Asp175) (Cell Signaling, 9664), Caspase-3 (Cell Signaling, 14220), Phospho-mTOR (Ser2448) (Cell Signaling, 5536), mTOR (Cell Signaling, 2983), Phospho-p70 S6 Kinase (Thr389) (Cell Signaling, 9234), Phospho-p70 S6 Kinase (Ser371) (Cell Signaling, 9208), Phospho-4E-BP1 (Thr37/46) (Cell Signaling, 2855), CDK2 (Cell Signaling, 2465), CDK6 (Cell Signaling, 3136), Cyclin D1 (Cell Signaling, 2978), CDK4 (Cell Signaling, 12790), DDR1 (Cell signaling, 5583), p-DDR1 (Y792) (Cell signaling, 11944).

After binding antigen-specific primary antibodies, the blots were incubated with a secondary antibody conjugated with horseradish peroxidase. To detect the signal, blots were incubated with Clarity Western ECL Substrate (Bio-rad, California, USA), and the chemiluminescent was detected by the ChemiDoc MP Imaging System (Bio-Rad).

### Apoptosis assay

The condition media and the cells are collected into a 15 mL tube. The cells are packed and resuspended with 0.5 mL cold PBS. After placing the cells on ice for 10 mins, we centrifuge the tube at 5000 rpm for 10 mins. The supernatant is removed, and 100 µL of cold 1X Binding Buffer, as well as 2 µL FITC-Annexin V and 2 µL propidium iodide (PI) (BD Biosciences, 556547, New Jersey, USA) are added. The sample is incubated at room temperature for 15 mins, incorporated with 400 μL of 1X Binding Buffer, and analyzed by flow cytometry.

### Cell viability assay

MPNST cell viability was determined by MTT (3-(4,5-dimethylthiazol-2-yl)-2,5-diphenyltetrazolium bromide) assay. The MTT stock solution was prepared by dissolving MTT powder (BioShop, Burlington, Canada) in PBS at a concentration of 2.5 mg/ml, then filtration through a 0.22-µm filter and stored at 4°C. For the MTT assay, the MPNST cells were cultured in a 96-well plate at a concentration of 3000-6000 cells/well, depending on the cell size and proliferation rate. The day after the treatment, 20 µl of MTT solution was added to each well of the sample. The MTT solution was incubated with the cells at 37°C for 1 hr and the supernatant was aspirated, followed by adding 100 µl DMSO to dissolve the crystals. The plate was carefully protected from light and shaken gently at RT for 20 min. The relative cell viability was calculated by the absorbance reading of 570 nm in a microplate reader.

### Immunofluorescent staining

MPNST cells were cultured and attached to a cover glass thinner than 0.17 mm. After DDR1-IN-1 treatment, cells were washed with PBS and fixed with 4% formaldehyde for 5 min, followed by PBS washes. Slides were blocked by 10% FBS for 30 mins at 4°C. The Phospho-MLKL (Ser358) (Cell Signaling, 91689) antibody was incubated with the slides at 4°C overnight followed by PBS washes. Alexa Fluor 488 conjugated secondary antibody was incubated for 1 hr at 4°C, followed by PBS washes. Fluoroshield mounting medium with DAPI was applied to mount the samples on a microscopic slide. Fluorescence imaging was performed using Zeiss AxioObserver Z1 Microscope.

### Acridine orange staining

MPNST cells were cultured, washed, and fixed as described in the immunofluorescent staining. After fixation, 1 µg/ml of acridine orange was incubated with the samples at dark for 10 min, followed by PBS washes. To remove the excess stain, we rinse the slide with PBS. The slides were mounted and imaged by Zeiss AxioObserver Z1 Microscope.

### General Statistics

The results were presented as the mean ± S.E.M. Each experiment was repeated independently at least three times unless indicated differently. The statistic was performed by unpaired two-sided t-test to ascertain differences between any two groups. A significance level of p < 0.05 was deemed significant, with all corresponding p values either depicted in figures or annotated in figure legends. Statistical computations were performed using GraphPad Prism 10 for Mac (GraphPad Software).

## Supplementary information

Supplementary Figures

Original WB

## Data Availability

The data presented in this study are available on request from the corresponding author.
