# Supplementary material for: Discoidin domain receptor inhibitor DDR1-IN-1 induces autophagy and necroptotic cell death in malignant peripheral nerve sheath tumor
Source: Cell Death Discov. 2025 Mar 1;11:83. doi: 10.1038/s41420-025-02367-2 (PMC11873111; doi:10.1038/s41420-025-02367-2)
Supplement: Supplementary file 1 — Supplementary Figures [file 41420_2025_2367_MOESM1_ESM.pdf]

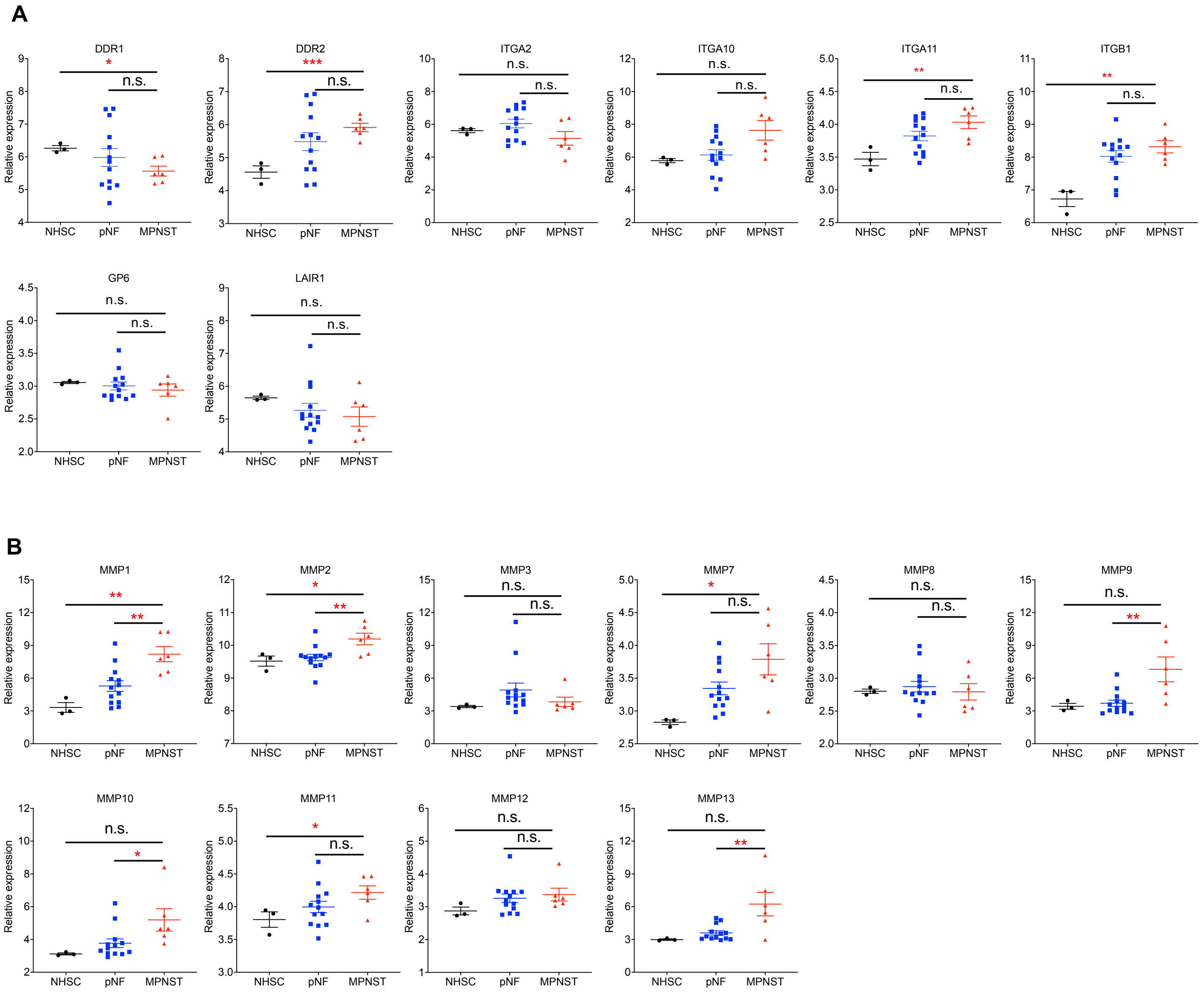

Supplemental Figure1. The expression level of collagen-related genes in clinical plexiform neurofibroma (pNF) and MPNST cases.

In GSE41747-10371, the RNA expression level of collagen receptors (A) and matrix metalloproteinases (B) in NHSC, pNF, and MPNST clinical cases were plotted, and the p-value was demonstrated. *P<0.05, **P<0.01, ***P<0.001, ****P<0.0001.

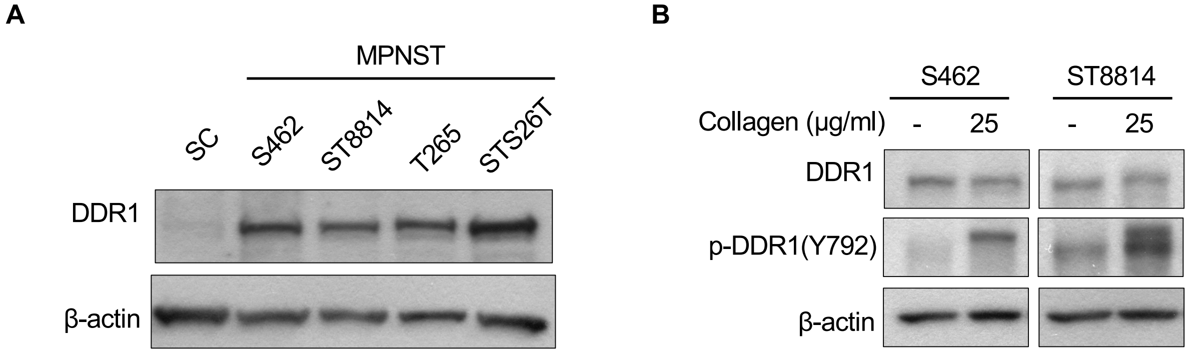

Supplemental Figure 2. Extracellular collagen-induced DDR1 signaling pathway activity in MPNST cells.
(A) The DDR1 protein expression level in Schwann cell (SC) and MPNST cell lines, S462, ST8814, T265, and ST26T, were evaluated through Western blotting. (B) MPNST cells were treated with or without 25 μg/mL collagen for 24 hours extracellularly. The levels of DDR1 and phosphorylated DDR1 (p-DDR1) were detected to elucidate the activity of the DDR1 signaling pathway.

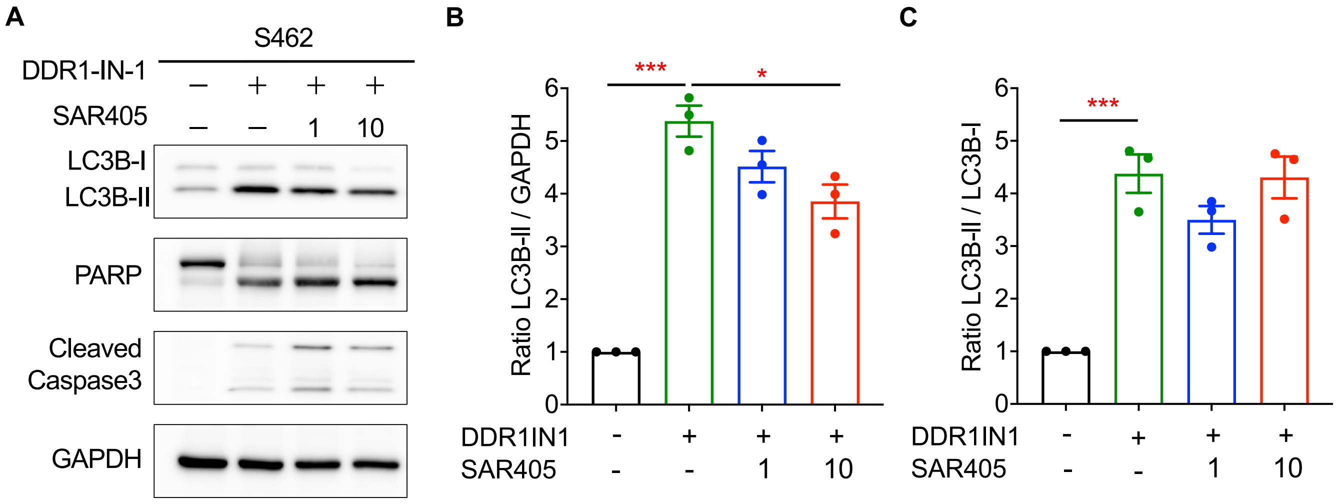

Supplemental Figure 3. SAR405 reversed the DDR1-IN-1-induced LC3B-II accumulation.
(A) Autophagy inhibitor, SAR405 (1 μM, 10 μM), was co-treated separately with 10 μM DDR1-IN-1 to S462 cells for 24 hours. Total protein was extracted, and the cell death-related markers were demonstrated through western blotting. (B) The ratio of LC3B-II to GAPDH was calculated and compared between each group (n=3), and (C) the ratio of LC3B-I to LC3B-II was calculated and compared between each group (n=3). The ratios were plotted in bar charts, and the p-values are shown. *P<0.05, **P<0.01, ***P<0.001, ****P<0.0001.

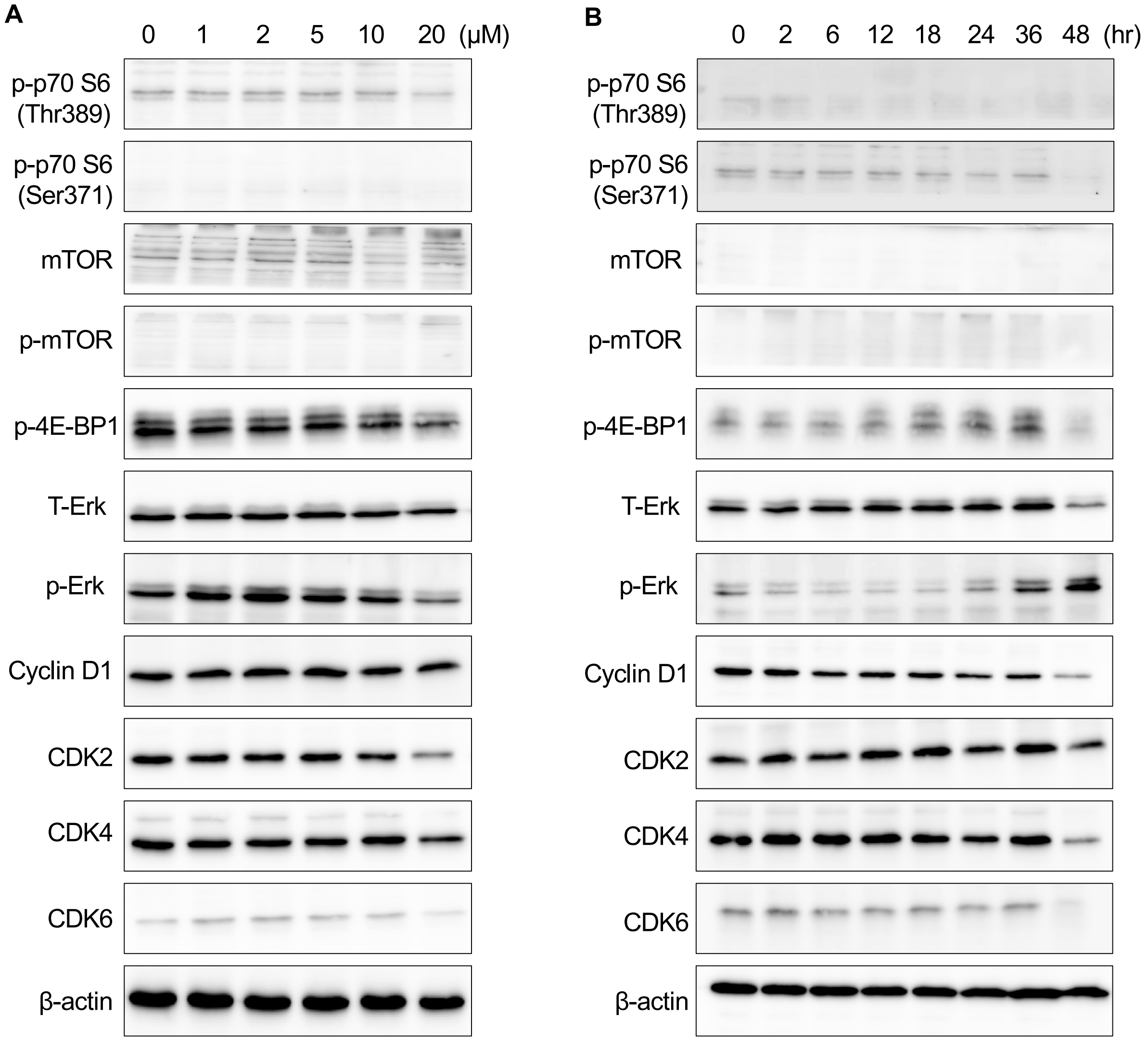

Supplemental Figure 4. DDR1-IN-1-induced MPNST cell death was not related to the cell proliferation signal. The MPNST cell line STS26T was treated with indicated concentrations of DDR1-IN-1. The protein expression was elucidated through Western blotting. Various markers involved in cell survival signals and cell cycle were detected. (A) STS26T MPNST cells treated with 0-20 μM DDR1-IN-1 for 24 hours, as well as (B) STS26T cells treated with 10 μM DDR1-IN-1 for 0-48 hrs, were extracted.

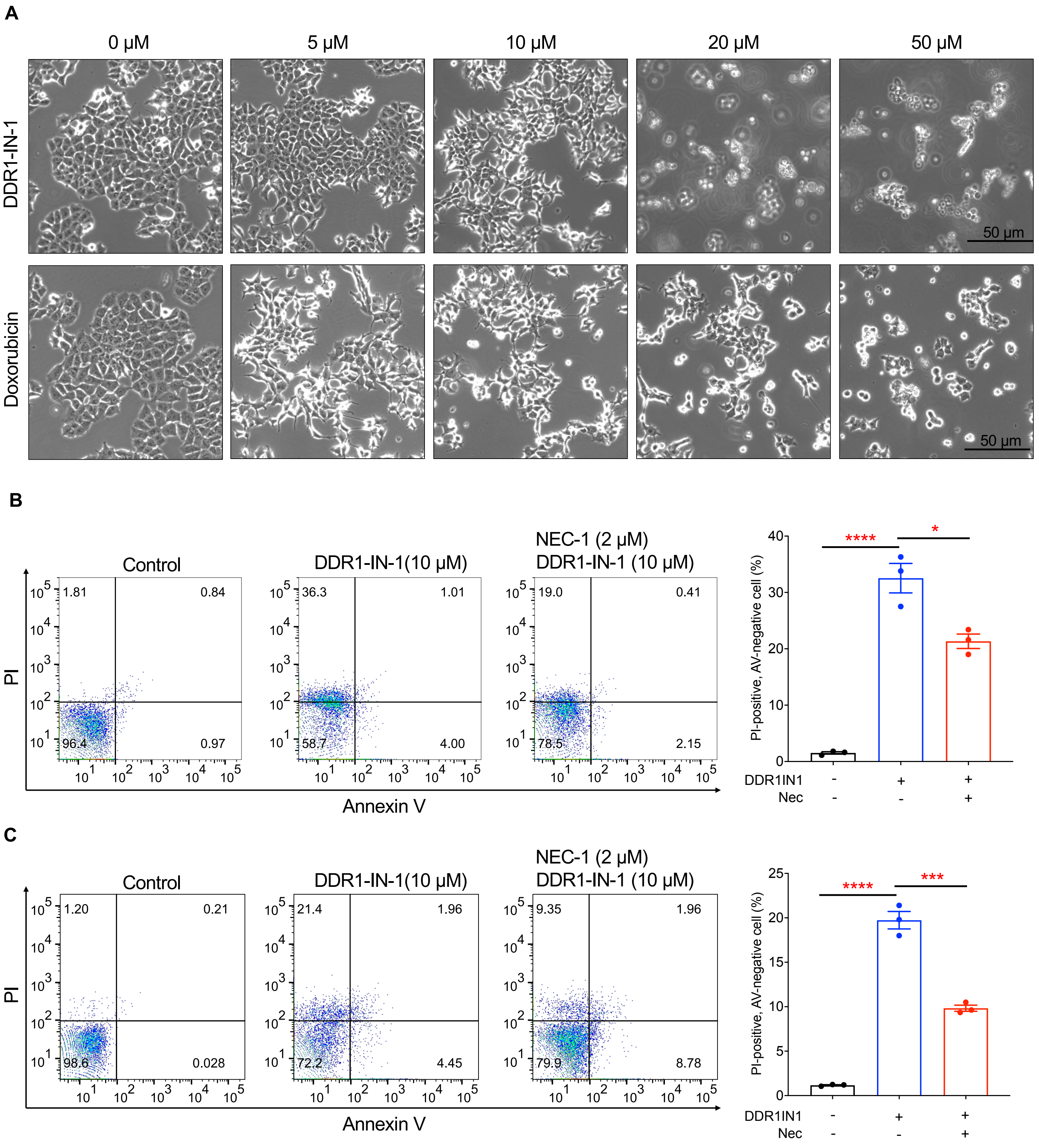

Supplemental Figure 5. DDR1-IN-1-induced necrotic cell death was not specific to MPNST.
(A) Images of MCF-7 cell line treated with indicated doses of DDR1-IN-1 and Doxorubicin for 24 hours were shown. AV/PI double staining was performed in (B) breast cancer MCF-7 cell line and (C) glioblastoma U-87 cell line. Cells were treated with either vehicle, 10 μM DDR1-IN-1, or 10μM DDR1-IN-1 combined with 2 μM NEC-1, a necroptosis inhibitor, for 24 hours. The cells were collected and classified through flow cytometry. The percentage of PI+/AV- cells was calculated and plotted in the bar chart, and the p-value was shown. * P<0.05, **P<0.01, ***P<0.001, ****P<0.0001.
