# Supplementary figures and images for: Discoidin domain receptor inhibitor DDR1-IN-1 induces autophagy and necroptotic cell death in malignant peripheral nerve sheath tumor
Source: Cell Death Discov. 2025 Mar 1;11:83. doi: 10.1038/s41420-025-02367-2 (PMC11873111; doi:10.1038/s41420-025-02367-2)

Figure 2

A

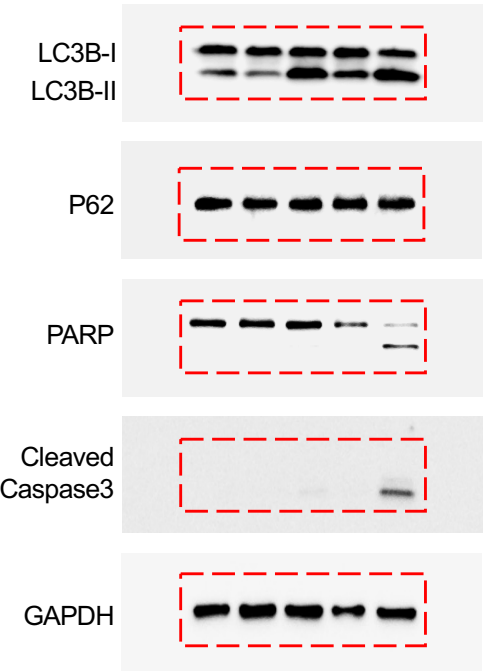

B

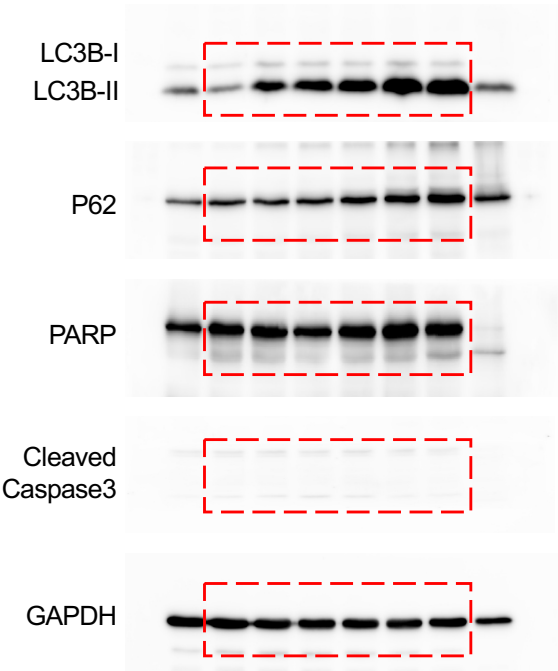

C

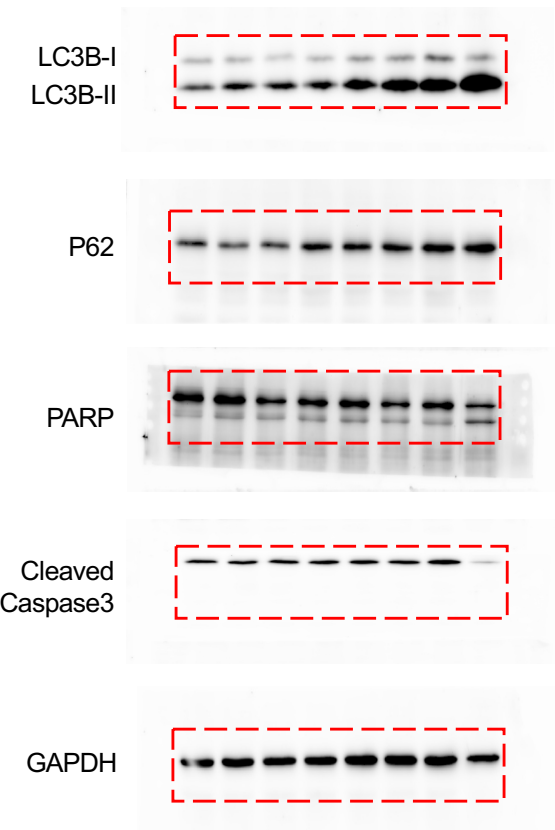

D

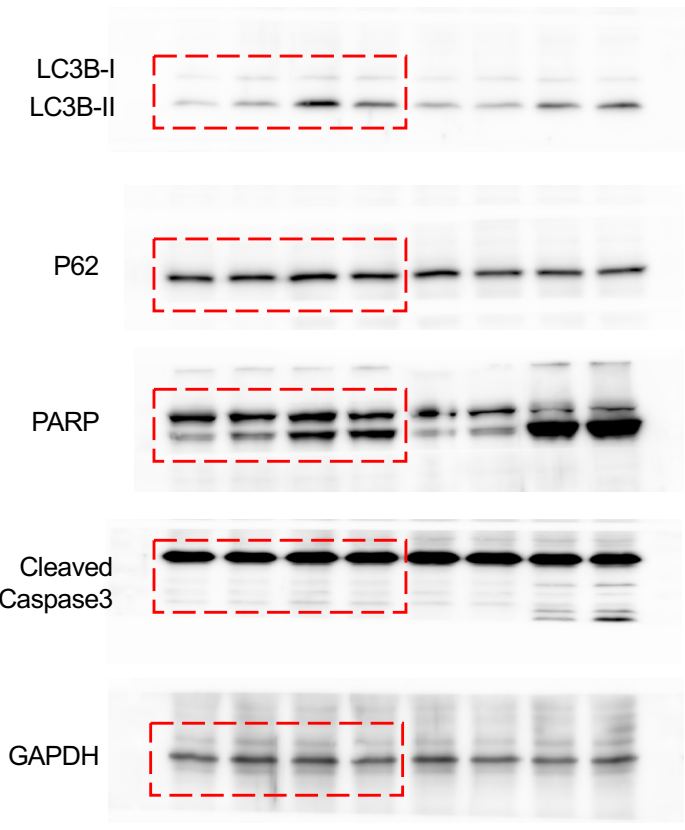

Figure 4

E

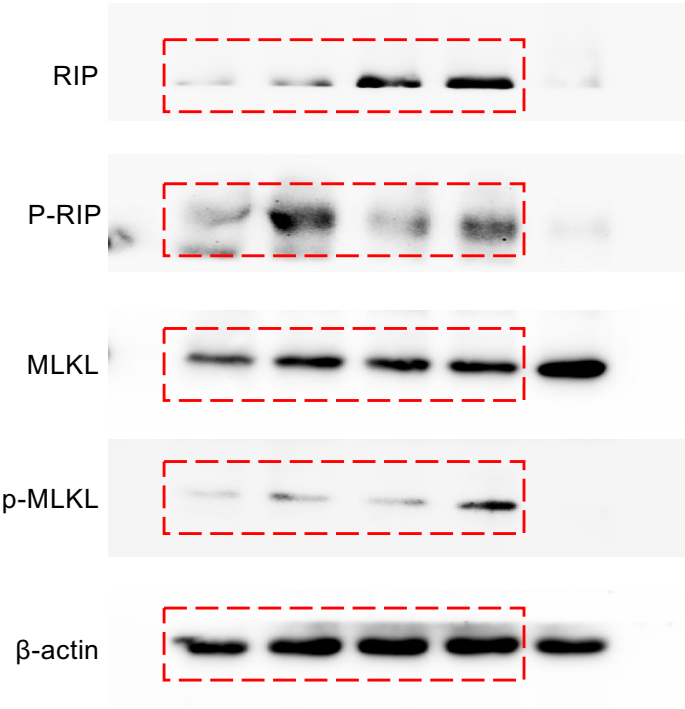

Supplemental Figure 2

**A**

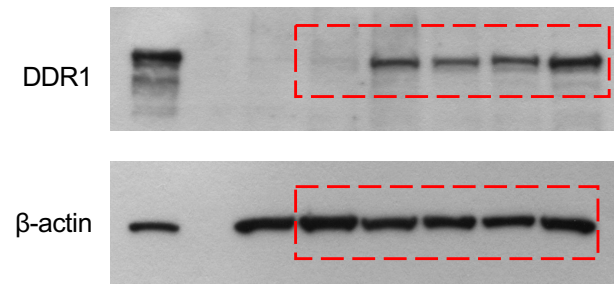

**B**

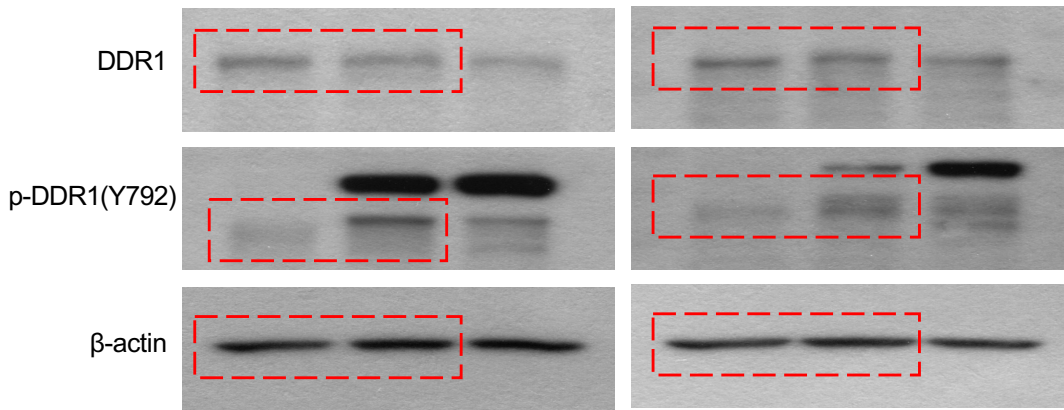

**A**

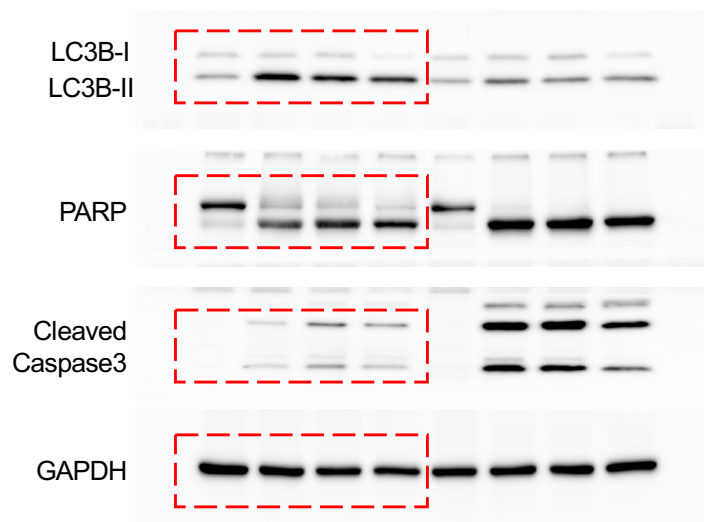

Supplemental Figure 4

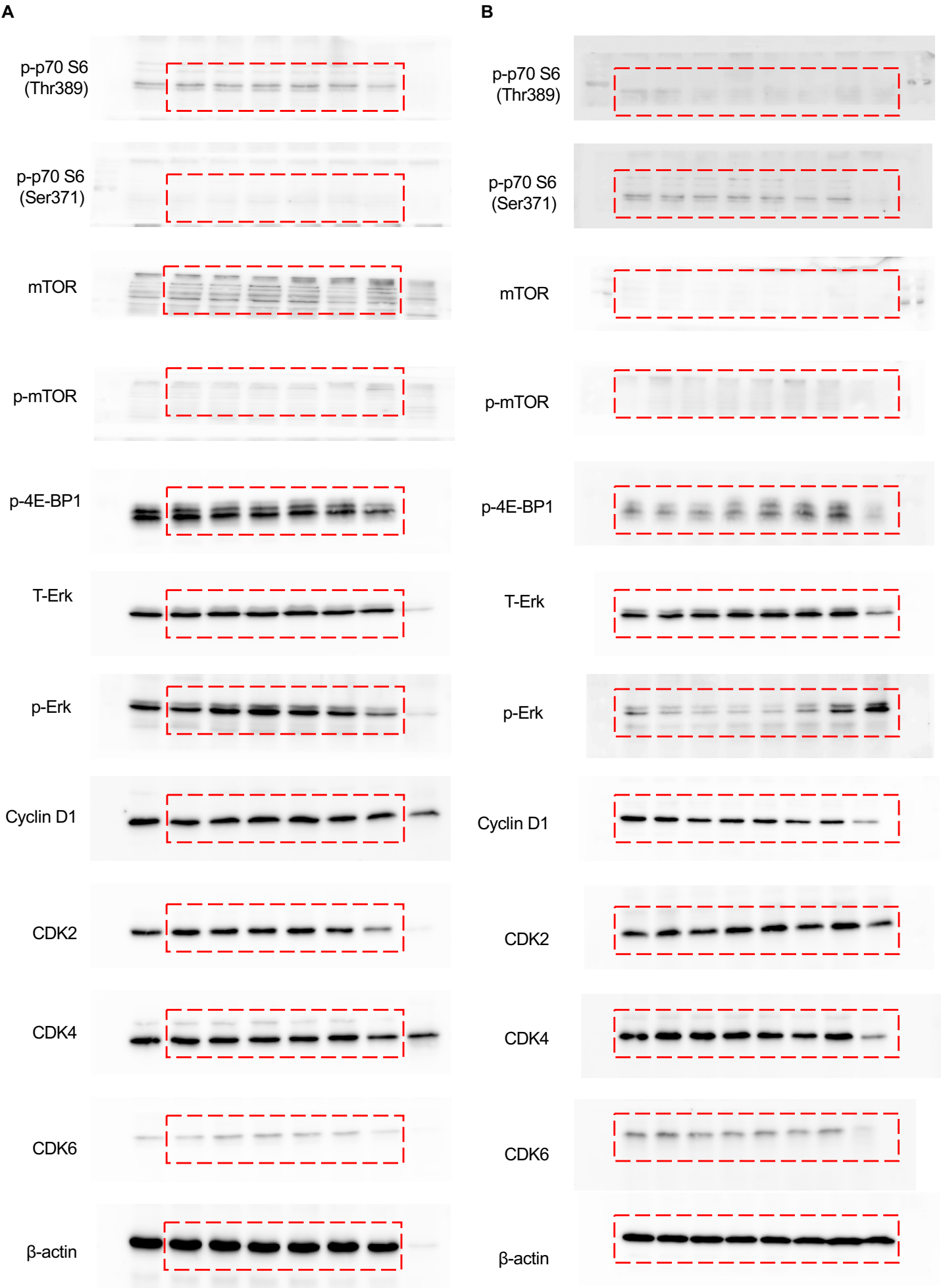

Supplement: Supplementary file 2 — Original WB [file 41420_2025_2367_MOESM2_ESM.pdf]
